# Differential DNA methylation at conserved non-genic elements and evidence for transgenerational inheritance following developmental exposure to mono(2-ethylhexyl) phthalate and 5-azacytidine in zebrafish

**DOI:** 10.1186/s13072-017-0126-4

**Published:** 2017-04-12

**Authors:** Jorke H. Kamstra, Liana Bastos Sales, Peter Aleström, Juliette Legler

**Affiliations:** 1 Faculty of Veterinary Medicine, Department of Basic Sciences and Aquatic Medicine, CoE CERAD, Norwegian University of Life Sciences, P.O. Box 8146 Dep., 0033 Oslo, Norway; 2 Institute for Environmental Studies, VU University Amsterdam, Amsterdam, The Netherlands; 3 Institute for Environment, Health and Societies, College of Health and Life Sciences, Brunel University London, Uxbridge, UK

**Keywords:** Phthalate, 5-Azacytidine, Epigenetics, DNA methylation, Transgenerational, Zebrafish, Toxicology, Environmental stress

## Abstract

**Background:**

Exposure to environmental stressors during development may lead to latent and transgenerational adverse health effects. To understand the role of DNA methylation in these effects, we used zebrafish as a vertebrate model to investigate heritable changes in DNA methylation following chemical-induced stress during early development. We exposed zebrafish embryos to non-embryotoxic concentrations of the biologically active phthalate metabolite mono(2-ethylhexyl) phthalate (MEHP, 30 µM) and the DNA methyltransferase 1 inhibitor 5-azacytidine (5AC, 10 µM). Direct, latent and transgenerational effects on DNA methylation were assessed using global, genome-wide and locus-specific DNA methylation analyses.

**Results:**

Following direct exposure in zebrafish embryos from 0 to 6 days post-fertilization, genome-wide analysis revealed a multitude of differentially methylated regions, strongly enriched at conserved non-genic elements for both compounds. Pathways involved in adipogenesis were enriched with the putative obesogenic compound MEHP. Exposure to 5AC resulted in enrichment of pathways involved in embryonic development and transgenerational effects on larval body length. Locus-specific methylation analysis of 10 differentially methylated sites revealed six of these loci differentially methylated in sperm sampled from adult zebrafish exposed during development to 5AC, and in first and second generation larvae. With MEHP, consistent changes were found at 2 specific loci in first and second generation larvae.

**Conclusions:**

Our results suggest a functional role for DNA methylation on cis-regulatory conserved elements following developmental exposure to compounds. Effects on these regions are potentially transferred to subsequent generations.

**Electronic supplementary material:**

The online version of this article (doi:10.1186/s13072-017-0126-4) contains supplementary material, which is available to authorized users.

## Background

Exposure to environmental stressors early in life, such as malnutrition, stress and chemical compounds has been hypothesized to play a role in the latent onset of diseases and adverse effects that may be transferred to subsequent generations [1]. In agreement with this ‘developmental origins of health and disease’ paradigm [2], a plurality of epidemiological and animal studies during the last decade have reported latent and transgenerational effects of developmental exposure to environmental stressors (reviewed in [3–5]). These latent and heritable effects may not be attributed to genetic variation and are suggested to be of an epigenetic nature [6]. DNA methylation and chemical modifications on histone tails are both considered epigenetic marks with high potential to be inherited and could therefore act as the drivers behind latent and transgenerational effects [7].

DNA methylation, by cytosine (mC), is dynamically regulated throughout life, particularly during early development. During mitosis, hemimethylated DNA in daughter cells is remethylated to the state of the mother cell, by maintenance of DNA methyltransferase 1 (DNMT1) [8]. Genome-wide reprogramming of DNA methylation takes place during both early zygotic development and the development of the gametes [8, 9]. From zygote to blastula stage, a wave of genome-wide demethylation ensures a totipotent cell state, followed by remethylation mediated by de novo DNMTs. A second wave of reprogramming follows during primordial germ cell (PGC) development, to ensure a gender-specific methylation state in gametes [10]. Recently, dynamic enhancer methylation during early developmental stages has been observed in vertebrates, linked to many developmental genes [11, 12]. Clearly, during these dynamic periods of epigenetic regulation, environmental stress targeted to the epigenome could potentially affect early embryonic development.

In this study, we used zebrafish as an alternative model to study transgenerational epigenetic inheritance. Zebrafish are a suitable vertebrate model in epigenetic studies, as they harbor similar methylation patterns compared to mammals and show conservation of DNA methylation and other epigenetic pathways [9]. However, there are distinct differences between the methylome of zebrafish and mammals. It is suggested that the paternal genome during zebrafish zygotic development is relatively resistant to demethylation [13], whereas recent research in mice suggests that the paternal genome is actively demethylated [14]. Also, in zebrafish, active developmental enhancers are hypermethylated, which has not been observed in other species [15]. Furthermore, the second wave of DNA methylation reprogramming in PGCs has not been confirmed in zebrafish. However, the methylome of sperm and oocytes in zebrafish differs significantly [16], which suggests that DNA methylation reprogramming events occur in zebrafish PGCs as well. Compared to mammalian models, zebrafish has the advantage that external exposures of eggs directly after fertilization is possible, thereby enabling the inclusion of both reprogramming events during exposures. Exposure of zebrafish embryos directly after fertilization means that the F0 generation is directly exposed, as well as the developing primordial germ cells which will ultimately become the F1. The F2 generation is the first completely unexposed progeny, as opposed to the F3 in mammalian studies [7].

Here, we examined the direct, latent and transgenerational effects of two model compounds with different modes of action on DNA methylation in zebrafish. We used mono(2-ethylhexyl)phthalate (MEHP), a major metabolite of di-2-(ethylhexyl) phthalate (DEHP), a high-volume production plasticizer ubiquitously present in the environment [17]. Developmental DEHP exposure has been associated with many health effects, such as reproductive toxicity, obesity and dyslipidemia [18–20], and its use in food contact materials, baby products and toys has been restricted in the European Union, although it is still allowed in electronic devices and medical equipment [21]. DEHP is rapidly metabolized to monophthalates, such as MEHP, and the toxicity of DEHP is considered to be mediated by MEHP rather than the parent compound [20]. Several studies have shown latent and transgenerational effects on DNA methylation in different tissues following in utero DEHP exposures in different rodent models, as a pure compound or in a mixture with other plasticizers [22–26]. One of these studies has shown an obese phenotype in rat offspring and linked differentially methylated regions (DMRs) to an obesity-related gene network [23]. In our study, we exposed zebrafish during early life stages (0–6 dpf) to the active metabolite MEHP, as gut and liver mediated metabolism of DEHP during these early stages may be limited. Furthermore, we exposed embryos to 5-azacytidine (5AC), a DNA methylation inhibitor used in the treatment of myelodysplastic syndrome and acute myeloid leukemia [27]. During the cell cycle, 5AC incorporates into DNA, irreversibly binds to and inactivates DNMT1, resulting in genome-wide hypomethylation [28]. As the hypomethylating properties of 5AC have been previously observed in zebrafish [29], we used this compound as a positive control, and as a proof of principle of transgenerational epigenetic inheritance following chemical exposure. To our knowledge, no previous studies assessing the transgenerational effects of early exposures to 5AC on the methylome have been carried out.

In this study, we assessed direct, latent and transgenerational effects of MEHP and 5AC using three different approaches. We analyzed global 5-hydroxymethyl-2′-deoxycytidine and 5-methyl-2′-deoxycytidine (hmC and mC) levels, genome-wide, and loci-specific mC levels, with liquid chromatography–tandem mass spectrometry (LC/MS), reduced representation bisulfite sequencing (RRBS) and amplicon bisulfite high-throughput sequencing (BisPCR2), respectively. Our data indicate genome-wide effects on DNA methylation following developmental exposure to both MEHP and 5AC on a multitude of loci, which were associated with specific biological pathways and enriched at conserved non-genic elements. A subset of DMRs were transgenerationally inherited in F2 larvae following both MEHP and 5AC exposures.

## Results

### Quality control

To assess whether developmental exposures to MEHP and 5AC altered mC and hmC at global levels, we used LC/MS analysis based on a method we recently developed [29]. Using internal standards to account for inter and intra experimental variation, we observed excellent reproducibility between experiments. Quality control DNA showed a relative standard deviation of 1.27 and 0.81% for hmC and mC, respectively (data not shown).

For RRBS analysis, we used a mapping pipeline specifically designed for RRBS data, developed by the Babraham Institute (Trim_galore and Bismark [30]). Using this pipeline, we were able to map around 59% of the sequences, generating an average of 444,855 analyzed Cs in CpG context per replicate with at least 10 reads (Additional file 1: Table 1). This is comparable to previously reported RRBS analysis in zebrafish brain and liver [31]. methylKit analysis estimates the bisulfite conversion efficiency using Cs in non-CpG context, which was very consistent between the samples, at an average of 99.2% (Additional file 1: Table 1).

Specific analysis of differentially methylated CpG sites (DMCs) was performed with a recently developed method, BisPCR2 [32]. To account for unforeseen biases, the method was thoroughly validated using a bisulfite converted standard curve of 0–100% methylated DNA to check for PCR bias and two samples that were analyzed in duplicate for technical variation. We were able to map >90% of the reads to the 10 loci covering a total of 103 CpG sites, with high accuracy between technical replicates (Additional file 1: Figure 1). Linear relationships were found at all loci analyzed, except for 4 CpG sites at Chr2:32025720, Chr2:32025757, Chr25:36706591 and Chr25:36706627, and these were excluded from the analyses (Additional file 1: Figure 2).

Finally, we validated our RRBS results against the BisPCR2 method. We were able to assess 49 mutually analyzed CpG sites between the two methods, which showed a high correlation (Spearman *r* = 0.889, *P* < 0.0001, Additional file 1: Figure 3), indicating that the results were consistent between the two methods.

## 5AC exhibits transgenerational phenotypic effects

**Fig. 1:**
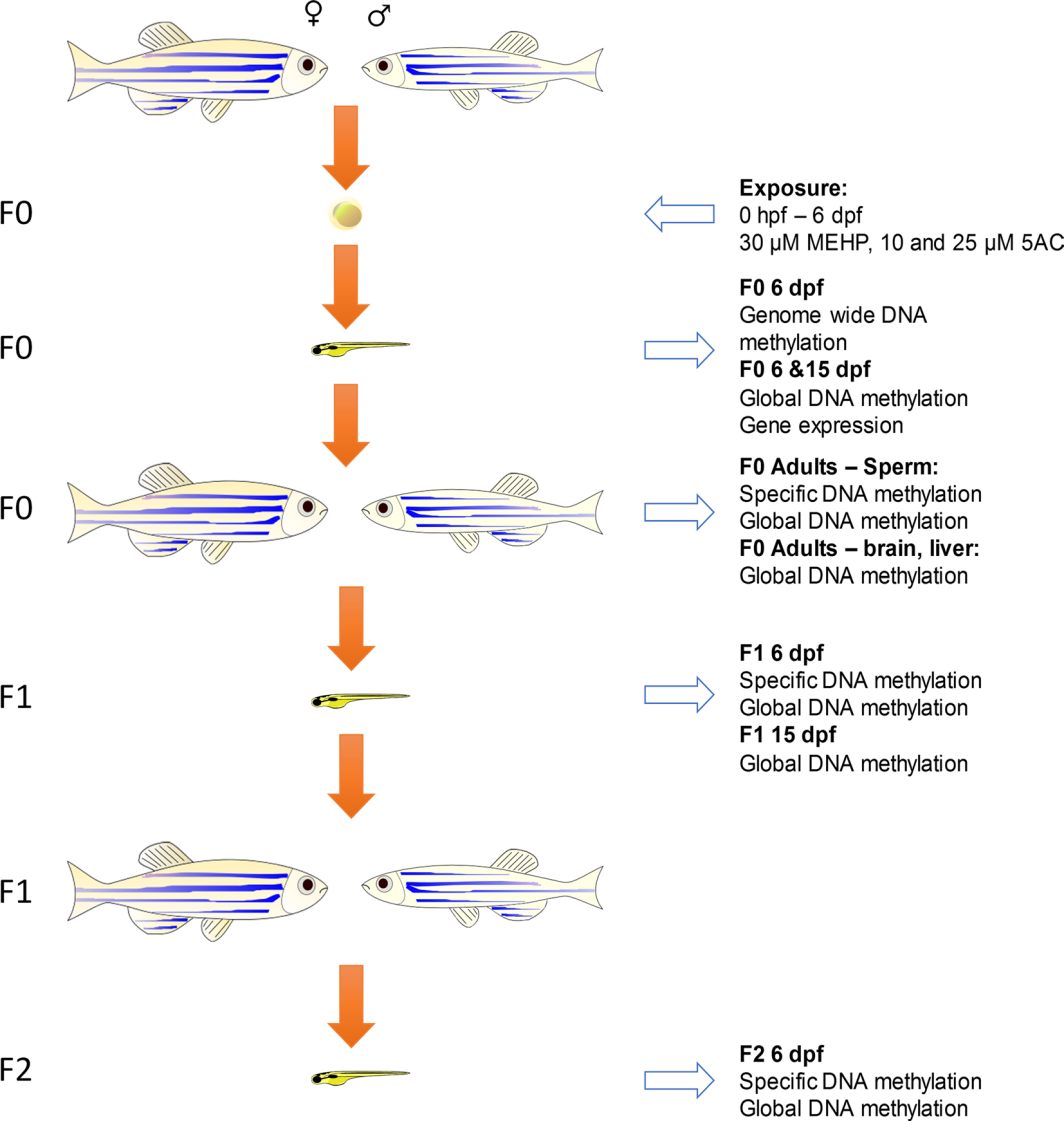
Setup of the transgenerational exposure experiment. Fish were exposed to mono(2-ethylhexyl) phthalate and 5-azacytidine (5AC) directly after fertilization up to 6 dpf. Adult F0 were in-crossed to generate F1 and F2. Analysis was performed at the different stages as indicated in Materials and methods. *hpf* hours post-fertilization, *dpf* days post-fertilization)

**Fig. 2:**
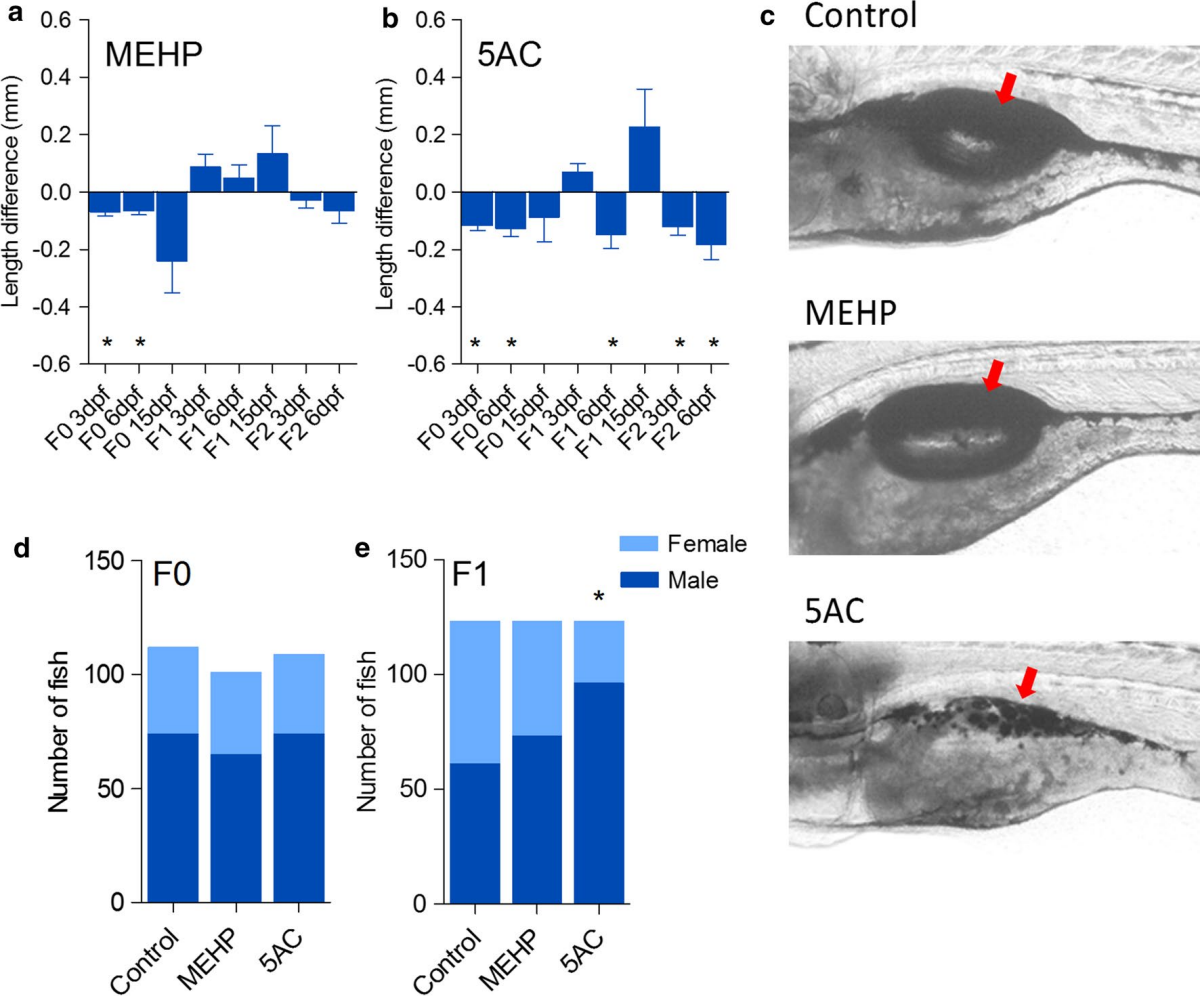
Phenotypic effects after developmental exposure of zebrafish (0-6 days post-fertilization) to mono(2-ethylhexyl) phthalate and 5-azacytidine (5AC). **a**, **b** Absolute difference in length of larvae compared to control (*error bars* represent SEM, *P < 0.05, two-tailed *t* test, with Bonferroni multiple comparison correction). **c** Representative images of effects on swim bladder inflation and intestinal development with 5AC exposures at 15 dpf. *Arrows* indicate swim bladder. **d**, **e** Sex ratios in F0 and F1 generations (*P < 0.001, Chi-square)

We exposed zebrafish embryos from 0 to 6 dpf and followed them up until adulthood. In-cross F1 and F2 generations were established, in which F2 is the first unexposed progeny (Fig. 1). We used non-embryotoxic concentrations of MEHP and 5AC (30 and 10 µM, respectively) that did not cause observable effects on developmental endpoints as defined by the standard zebrafish embryo toxicity assay [33]. In addition to 10 µM, embryos exposed to 25 µM 5AC from 0 to 6 dpf were included as a positive control for global hypomethylation [29]. Significant effects were observed on F0 larval length with both MEHP and 5AC exposure at 3 and 6 dpf (Fig. 2a, b). A transgenerational effect was observed on larval length in F1 and F2 exclusively for the 5AC exposure, which was most pronounced at 6 dpf (Fig. 2b).

A clear effect on swim bladder inflation and abnormal intestinal development was observed in 5AC treated F0 fish at 15 dpf (Fig. 2c and Additional file 1: Figure 4). However, no larval lethality was found and fish grew to adulthood without apparent effects. Additionally, the effects on intestine and swim bladder were not observed in F1 (data not shown). We observed a significant shift in gender toward males in the F1 generation with 5AC, but not after MEHP exposures (Fig. 2d, e).

## 5AC and MEHP affect *dnmt* gene expression and global mC and hmC levels

**Fig. 3:**
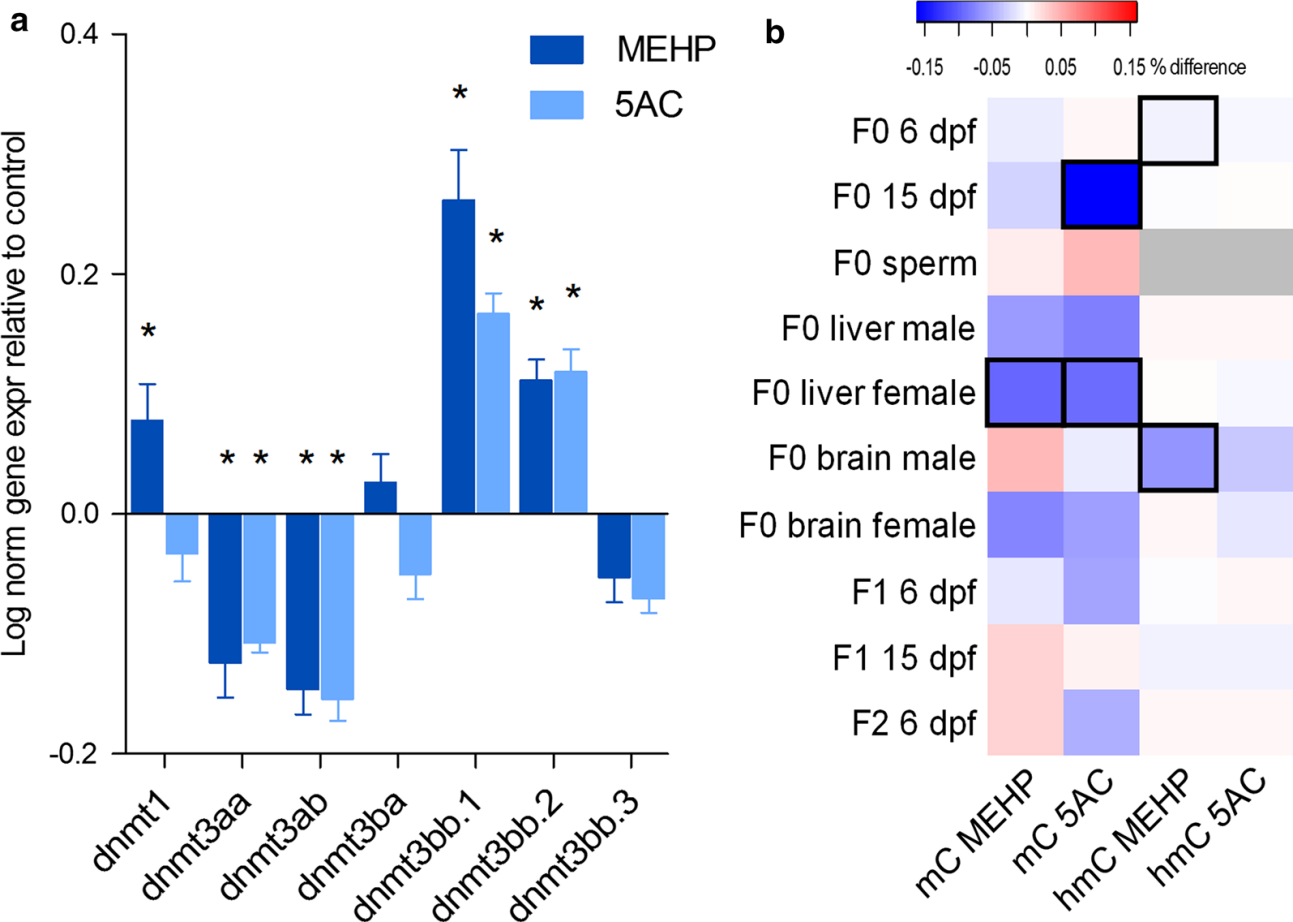
Gene expression analysis of *dnmt* variants and global methylation. **a** Log-normalized gene expression relative to control in F0 6 days post-fertilization larvae (*Error bars* represent SEM, *P < 0.05, ANOVA with Dunnets post hoc test). **b** Heat map of absolute differences in global cytosine methylation and cytosine hydroxymethylation compared to control. *Black squares* indicate significant changes relative to control (*P* < 0.05, two-tailed *t* test, using Bonferroni multiple comparisons correction). *Gray* indicates non-detectable levels

We assessed DNA-methyltransferase (*dnmt)* gene expression of all 3 *dnmt* orthologues and their respective paralogues in F0 larvae at 6 dpf (Fig. 3a). Dnmt1 is mainly involved in maintenance of DNA methylation during cell replication, whereas the other 6 genes encode de novo Dnmts, which are suggested to have both tissue- and promoter-specific functions [9]. Significant upregulation of *dnmt1* was observed with MEHP exposure, but not with 5AC (Fig. 3a). Both exposures show very similar differential expression profiles for the de novo *dnmt3* orthologues, where *dnmt3aa* and *dnmt3ab* paralogues are downregulated and *dnmt3bb.1* and *dnmt3bb.2* paralogues are upregulated. The differential expression profiles of the *dnmts* indicate interference in DNA methylation pathways with both exposures.

We observed a significant decrease in global mC levels at 25 µM 5AC in 6 dpf F0 larvae DNA (Additional file 1: Figure 5). Additionally, a decrease in global mC levels was observed at 15 dpf F0 following 10 µM 5AC exposures (Fig. 3b). Global demethylation was also observed in livers from adult females exposed to both MEHP and 5AC (Fig. 3b). Decreased hmC levels following MEHP exposure were observed at 6 dpf F0 and in brain tissue of male fish, whereas no changes were observed on mC levels (Fig. 3b).

### RRBS reveals enrichment of DMRs on conserved non-genic elements

**Fig. 4:**
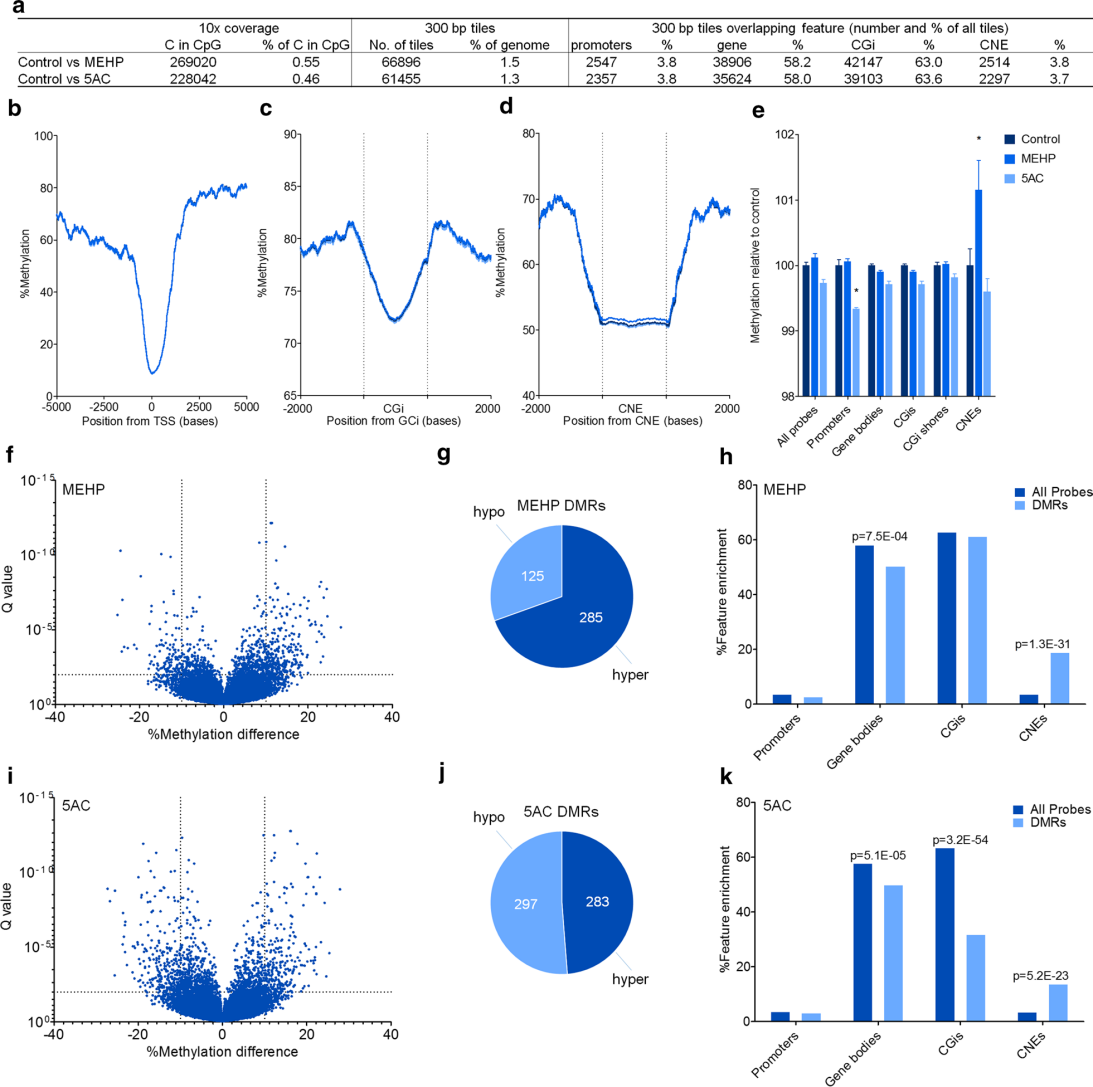
Reduced representation bisulfite sequencing (RRBS) results following developmental exposure to mono(2-ethylhexyl) phthalate (MEHP) and 5-azacytidine (5AC). **a** General statistics of RRBS analyses, showing mutual analyzed Cs in CpG context at a read depth of 10, number of tiles analyzed with regional analysis and the number of tiles overlapping different features. **b**–**d** Methylation profile spanning transcriptional start sites (TSSs), CpG islands and zebrafish conserved non-genic elements (zfCNEs). **e** Methylation levels relative to control overlapping different features. *Error bars* represent SEM; significance was calculated with two-way ANOVA, using Bonferroni multiple comparisons correction (*P* < 0.05). **f** Volcano plot of the methylation difference between MEHP and control. **g** Number of MEHP hypo and hyper differentially methylated regions (DMRs). **h** Enrichment plot of all tiles and DMRs overlapping different features. Significant enrichment calculated using a hypergeometric test. **i** Volcano plot of the methylation difference between 5AC and control. **j** Number of 5AC hypo and hyper DMRs. **k** Enrichment plot of all probes and DMRs overlapping different features. Significant enrichment calculated using a hypergeometric test

With RRBS, we were able to analyze around 200,000 mutually measured Cs (read depth >10) and over 60,000 mutually measured 300-bp tiles (Fig. 4a). Global methylation changes by RRBS in features as promoter regions (2000-bp upstream of transcriptional start sites) (TSSs), CpG islands (CGis) and shores and gene bodies were assessed using 300-bp tiles (Fig. 4a). A sharp decline in methylation around both TSSs and CpG islands was observed, with no apparent difference between the treatments (Fig. 4b, c). Overlap of tiles to a computationally derived list of conserved non-genic elements in zebrafish (zfCNEs) [33] also showed a general decline in methylation, but a distinct difference was found with MEHP exposures, which showed hypermethylation compared to control and 5AC samples (Fig. 4d). Calculation of the average methylation relative to the control over the different features revealed a significant decrease in methylation between control and 5AC at promoter regions and a significant increase between control and MEHP at zfCNEs (Fig. 4e).

methylKit analysis revealed 410 DMRs in F0 6 dpf larvae following exposure to MEHP, with a cutoff of 10% methylation difference and a *Q* value <0.01 (Fig. 4f). From these DMRs, more hypermethylated regions (70%) were observed than hypomethylated (Fig. 4g). When we mapped the MEHP DMRs to different genomic features, we observed an enrichment of DMRs at zfCNEs (Fig. 4h and Additional file 1: Table 2A, *P* = 1.3E−31). Additionally, limited overlap was found on gene bodies which indicates that DMRs are predominantly located outside gene bodies (Fig. 4h and Additional file 1: Table 2A, *P* = 7.5E−4). With 5AC, 580 DMRs were found with equal numbers of hyper and hypomethylated regions (Fig. 4i, j). As with MEHP, enrichment was found on zfCNEs (*P* = 5.2E−23) and limited overlap on gene bodies (*P* = 5.1E−05) (Fig. 4k and Additional file 1: Table 2B). Additionally, 5AC-specific DMRs had limited overlap at CGis (*P* = 3.2E−54) (Fig. 4k and Additional file 1: Table 2B). For both 5AC and MEHP exposures, we calculated that 44% of the DMRs overlapped with the 23% zfCNEs that are conserved with human and mice. We also analyzed developmental enhancer regions, as indicated by histone H3K4me1 and H3K27Ac marks, but could not find any enrichment of DMRs on these specific sites (data not shown) [34].

### DMR-associated genes are involved in several developmental and disease-related pathways

Next, we were interested to find out whether these DMRs are associated with specific biological pathways. Therefore, the Genomic Regions Enrichment of Annotations Tool (GREAT) was used to associate the DMRs with genes, which we subsequently imported into Ingenuity Pathway Analysis (IPA). We analyzed all DMRs, hypermethylated DMRs, hypomethylated DMRs and zfCNE-specific DMRs. With both exposures, pathways involved in transcriptional and developmental processes were enriched, such as pathways involved in pluripotency, TGF-β, Gα 12/13, Wnt/β-catenin (Table 1). Upstream regulators involved in development, such as SSH, TGF-β1, SOX2, POU5F1, were also enriched with both exposures, which could be a specific response to the toxicological stress of the compounds (Additional files 3 and 4).

**Table 1:** Top canonical pathways after ingenuity pathway analysis of all differentially methylated region-associated genes in zebrafish larvae exposed from 0-6 days post-fertilization to mono(2-ethylhexyl) phthalate (MEHP, 30 µM) and 5-azacytidine (5AC, 10 µM)

Canonical pathway	MEHP (*P* value)	5AC (*P* value)
Human embryonic stem cell pluripotency	7.36E−05	1.92E−05
Factors promoting cardiogenesis in vertebrates	2.32E−03	1.32E−06
TGF-β signaling	8.43E−06	6.28E−04
Wnt/β-catenin signaling	7.10E−06	8.00E−04
Axonal guidance signaling	2.68E−03	1.05E−05
Regulation of the epithelial–mesenchymal transition pathway	1.87E−05	4.91E−03
Gα12/13 signaling	3.59E−06	4.21E−02
Adipogenesis pathway	5.23E−03	1.17E−03
Role of NANOG in mammalian embryonic stem cell pluripotency	6.56E−03	9.54E−04
Epithelial adherent junction signaling	6.66E−04	1.93E−02

**Fig. 5:**
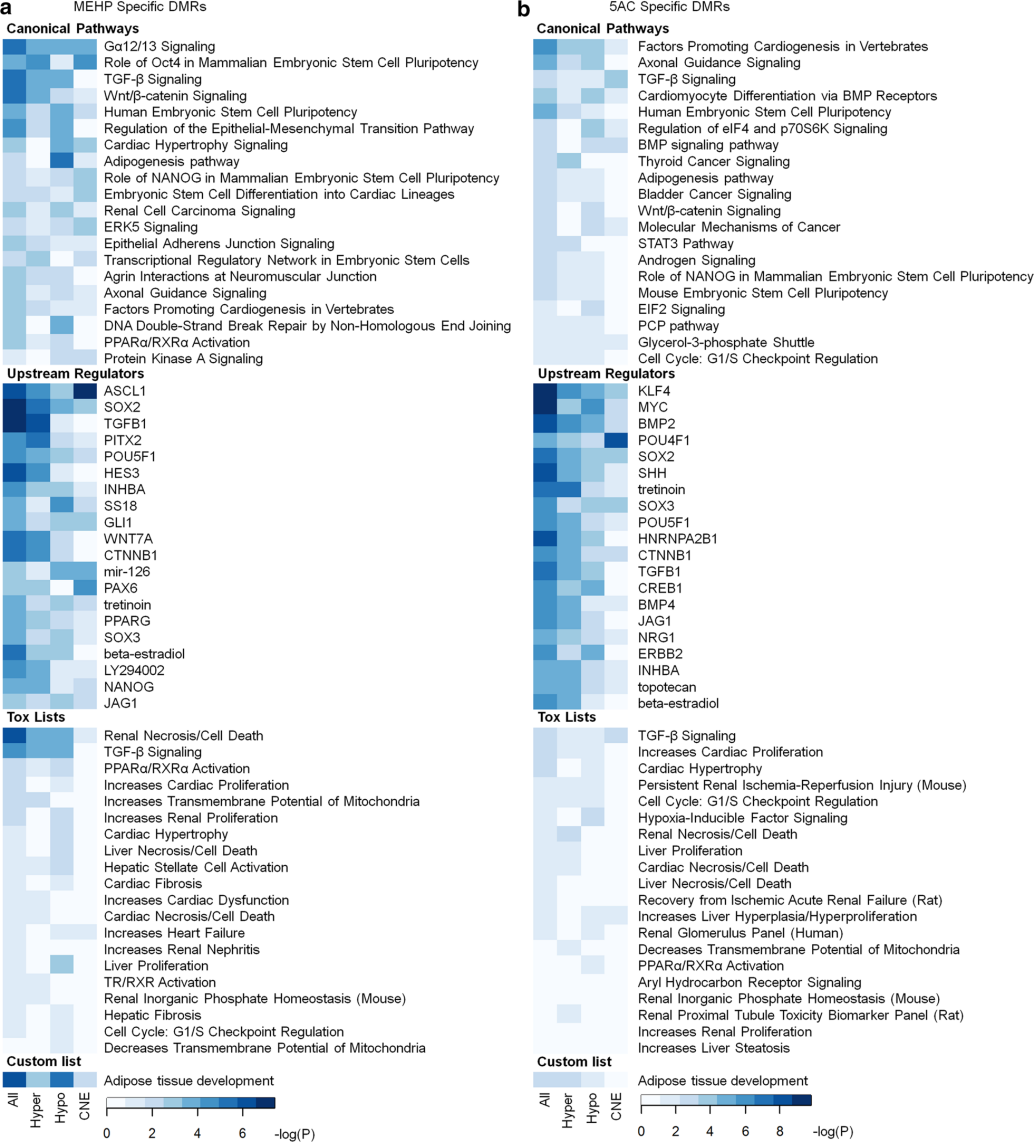
Ingenuity pathway analysis of mono(2-ethylhexyl) phthalate (MEHP) and 5-azacytidine (5AC)-specific differentially methylation regions (DMRs). Heat maps of −log(P) values for DMR-associated genes of **a** MEHP and **b** 5AC exposures. Heat maps show top 20 lists of predicted canonical pathways, upstream regulators, toxicology-related gene lists (Tox) of all (All), hypermethylated (hyper) and hypomethylated (hypo) DMRs, and DMR-specific zebrafish conserved non-genic elements (zfCNE). A custom imported list was also added (adipose tissue development). Extended lists are found in Additional file 3 and 4

Figure 5 presents heat maps of the compound-specific top 20 lists of canonical pathways, upstream regulators and toxicological lists, supplemented with a custom imported list (adipose tissue development). For MEHP, pathways and upstream regulators involved in adipogenesis and neuronal development were the most prominently enriched pathways (Fig. 5a). Involvement of the adipogenesis pathway was especially enriched in hypomethylated DMRs. Other upstream regulators, such as PPARG, PPARα/RXRα, TGFB1 and WNT7a, together with the custom adipogenesis list, also predicted involvement of MEHP in adipogenesis. Processes involving axonal guidance signaling, together with upstream regulators involved in neuronal development (ASCL1, SOX2, PAX6 and GLI1) of which some were specifically enriched at zfCNEs and hypermethylated DMRs, point toward disruption in nervous system development (Fig. 5a).

5AC-specific predicted pathways were involved in development and expression control of genes, such as KLF4, POU4F1, SHH, SOX3 (Fig. 5b). Also, as with MEHP, neuronal development may be impaired, as indicated by the enrichment of axonal guidance signaling pathways. Upstream regulators involved in (sensory) neuronal development were enriched (SOX2, SOX3, POU4F1) (Fig. 5b). Notably, POU4F1 was specifically enriched at zfCNEs (Fig. 5b). Furthermore, IPA analysis predicted effects on gastrointestinal diseases (*P* values of 6.25E−4–4.64E−13) and showed a strong enrichment in the upstream regulator HNF4a (*P* = 2.49E−6), a transcription factor known to be involved in gastrointestinal development (Additional file 4). Notably, development of body axis was among the most significant enriched lists in diseases and bio functions (*P* = 7.35E−17, Additional file 4), which is consistent with the effects found on body length following exposure to 5AC. 5AC did not seem to affect general toxicological pathways, since only weak enrichments were found in toxicological lists.

### Transgenerational effects observed with BisPCR2

**Fig. 6:**
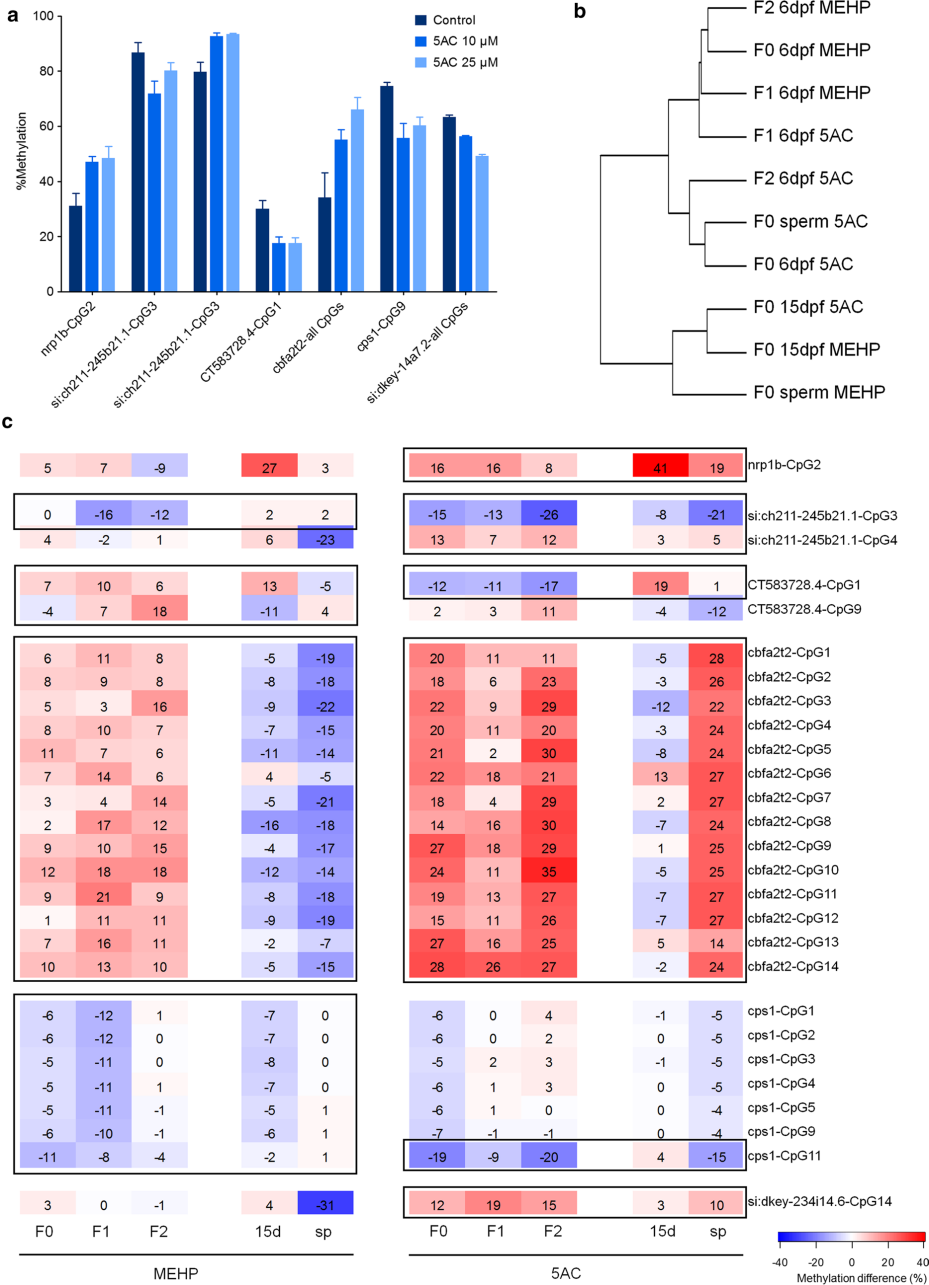
Locus-specific methylation analysis of different larval stages and sperm over generation following exposure to mono(2-ethylhexyl) phthalate (MEHP) and 5-azacytidine (5AC). **a** Loci showing significant effects with both 10 and 25 µM 5AC exposures compared to control at F0 6 dpf.* Error bars* represent SEM. **b** Hierarchical clustering of generational effects on DNA methylation differences of MEHP and 5AC exposures at 6 and 15 dpf and in sperm from F0 compared to their respective generational or tissue-specific controls (ward clustering). **c** Heat map showing the methylation difference compared to control of all CpG sites over generations (F0, F1 and F2) and 15 dpf (15) and sperm (sp) with at least one significant differentially methylated CpG, exhibiting a methylation difference of more than 10% as indicated by the *black squares*. Each stage or tissue-specific sample was compared with the controls from the same stage

Regions around ten specific DMCs were selected from the RRBS analysis that exhibited a difference in methylation larger than 20%. As nomenclature for the analyzed loci, we used the nearest annotated genes (Table 2). We analyzed 6 dpf F0, F1 and F2 larvae, as well as samples from 15 dpf F0 larvae and sperm from F0 fish, each with their respective control. Additionally, we included 25 µM 5AC exposures (6 dpf) in this analysis in order to investigate CpG site-specific dose-dependent relationships. These relationships were clearly visible following cluster analysis, with clusters of high methylation showing a decrease in methylation with the higher concentration and clusters of low methylation increasing with higher-concentration 5AC (Additional file 1: Figure 6). A number of these sites showed significant effects with both concentrations, with some showing a clear dose response (Fig. 6a).

**Table 2:** Overview of the 10 F0 loci analyzed with BisPCR2

Location	Nearest gene	CpGs analyzed
chr2:32025472–32025772	*mycb*	7
chr2:43611512–43611846	*nrp1b*	4
chr3:48276549–48276951	*si:ch211*–*245b21.1*	10
chr4:53831094–53831415	*CT583728.4*	14
chr6:57641533–57641533	*cbfa2t2*	14
chr9:39356640–39356640	*cps1*	11
chr20:43514527–43514862	*si:dkey*–*14a7.2*	10
chr12:29105587–29105587	*gabrz*	12
chr21:20158921–20158921	*si:dkey*–*247m21.3*	7
chr25:36706556–36706894	*si:dkey*–*234i14.6*	14

Hierarchical clustering of methylation levels at specific loci, as determined by BisPCR2 analysis, revealed that samples from F0 sperm and 15 dpf larvae clustered distinctively from 6 dpf samples (Additional file 1: Figure 7). This age-specific difference in methylation pattern was also apparent when looking at methylation after compound exposure, where methylation differences compared to control for MEHP at 15 dpf and sperm as well as 5AC at 15 dpf clustered distinctively from the 6 dpf samples (Fig. 6b). When focused on the difference between controls and exposed samples at all stages and F0 sperm, methylation patterns for MEHP at F0, F1 and F2 cluster together, as well as for 5AC at F0 and F2 (Fig. 6b), indicating that methylation changes caused by developmental exposure to these compounds in F0 are persistent from one generation to the next.

While the largest effects on methylation after BisPCR2 analysis were generally observed with 5AC exposures, we found significant effects over two generations following exposure to both compounds, in 2 and 6 out of 10 loci for MEHP and 5AC, respectively (Fig. 6c). With MEHP, a transgenerational increase in methylation was observed at the entire *cbfa2t2* locus, averaging from 6.8% in F1 to 11.6 and 10.7% in F1 and F2, respectively. Interestingly opposite effects were observed at 15 dpf and sperm, where methylation was decreased. MEHP-specific effects were observed at the *CT583728.4* locus up to F2 at CpG1 and CpG9, and F0 and in F1 at all CpGs at the *cps1* locus, but not F2. For 5AC, a strong transgenerational effect at the *cbfa2t2* locus was found, with an average regional increase in methylation up to 25% in F2 compared to control. Furthermore, transgenerational effects were observed at specific CpG sites at *nrp1b*-CpG2 (hyper), *si:ch211*-*245b21.1*-CpG3 (hypo), *si:ch211*-*245b21.1*-CpG4 (hyper), *CT583728.4*-CpG1 (hypo), *si:dkey*-*234i14.6*-CpG14 (hyper). At the *cps1* locus, a regional hypomethylating effect is present at F0, but not propagated to F1 and F2; however, a transgenerational effect is observed at CpG11.

## Discussion

In this study, we used next-generation sequencing to analyze DNA methylation on a genome-wide scale using zebrafish as an alternative model, in order to detect regional and site-specific changes following exposures to MEHP and 5AC. Our *dnmt* gene expression data and global methylation approach confirmed that both compounds interfered with DNA methylation pathways. RRBS analysis allowed us to link DMRs to specific pathways and aided in the prediction of adverse effects of these compounds. With the use of loci-specific bisulfite sequencing, we detected differentially methylated sites that persisted over two generations with both MEHP and 5AC exposures. We show that the combination of genome-wide analysis, followed by loci-specific analysis of newly discovered DMRs in subsequent generations, provides important insights in DNA methylation changes involved in transgenerational effects of developmental exposure to xenobiotic compounds.

To our knowledge, we show for the first time that developmental exposure to compounds specifically targets DNA methylation at conserved non-genic regions. We used a computationally derived list of zfCNEs which contained over 54,000 regions [35]. CNEs are generally located outside genic regions and can have cis-regulatory functions such as enhancers or silencers [36]. The zfCNEs are conserved in many species (at least three species per region), and have a 22% overlap with mice and human CNEs [35]. Furthermore, these regions show about 23% overlap with empirically derived developmental enhancer regions (H3K4me1 and H3K27Ac [34]), indicating a significant role for zfCNEs in early development. However, no enrichment was observed on developmental enhancer regions in this study, suggesting a regulatory function for zfCNE-specific DMRs outside developmental enhancers. Notably, DMRs were generally found outside gene bodies and outside CpG islands (specifically for 5AC), and were not enriched at promoter regions which indicates that DMRs are located at distal regulatory sites. A recent study summarizing DMRs derived from several transgenerational studies in rats exposed to different classes of compounds found an overrepresentation of DMRs at low CpG content areas, from both somatic tissues as sperm-specific DMRs [37]. Although we did not observe such effects with MEHP, 5AC-specific DMRs were over represented outside CpG islands. Further research to elucidate the functional and phenotypical significance of differential DNA methylation on these conserved elements is warranted. The application of novel methods using clustered regularly interspaced short palindromic repeat-CAS9 (Crispr/CAS9) engineered with de novo methylation and demethylation catalytic domains would be useful to target these specific regions and shed light on the functional significance of induced changes in DNA methylation [38, 39].

5-Azacytidine belongs to the family of azanucleosides, which are known cytotoxic and teratogenic agents, of which 5AC is the least toxic derivate [40]. We exposed embryos to 5AC at concentrations that were below the effect concentration in zebrafish embryo toxicity tests based on our own results and others [29, 41, 42]. Nevertheless, developmental exposure of zebrafish embryos to 5AC resulted in transgenerational effects on larval body length as well as direct effects on gastrointestinal development in F0 larvae at 15 dpf. IPA analysis revealed enrichments of genes involved in gastrointestinal diseases as well as HNF4a regulation. HNF4a is known to be involved in liver and intestinal development and is in combination with CDX2 crucial for columnar cell formation [43]. Interestingly, loss of columnar cells formation after *dnmt1* knockdown in zebrafish has been observed previously [44]. Additionally, we found direct and transgenerational effects of 5AC exposure on the *cbfa2t2* gene body. Effects on intestinal development and secretory cells formation in the small intestine have been reported for CBFA2T2^−/−^ mice, which also exhibit smaller phenotypes [45]. Although further research is necessary, our results point to a role for DNA methylation in intestinal development via Hnf4a signaling and suggest that the regional change in *cbfa2t2* methylation could be an interesting target.

MEHP-specific DMRs could be linked to genes that are involved in diseases known to be associated with this compound, in particular pathways related to obesity, mostly found on hypomethylated DMRs. MEHP is known to exert its adipogenic action via peroxisome proliferator-activated receptors (PPARs) [18]. IPA analysis revealed significant enrichment in upstream regulation of both PPARγ and PPARα and enrichments in upstream regulators TGFβ and WNT7a, all involved in adipogenic processes. The significant enrichment of the adipose tissue development gene list together with the enriched prediction molecules, cyclic AMP and dexamethasone and isobutylmethylxanthine, essential factors in the stimulation of adipogenic differentiation, implies a strong role of MEHP in adipogenesis. However, no effects were found on adipocyte differentiation in vivo (Bastos-Sales et al., unpublished results), suggesting that DMRs related to adipogenesis after direct exposure do not persist in adulthood. Furthermore, enrichments of upstream regulators as SOX2 and ASCL1 point to neurodevelopmental effects following MEHP exposure. Some of these upstream regulators are specifically enriched at zfCNEs, suggesting a regulating role of these conserved elements on neuronal development. Interestingly, the increased mRNA expression of *dnmt3bb.1* and *2*, known to be involved in brain development [46], coincides with hypermethylation of these genes at zfCNE DMRs.

Transgenerational effects with persistent differential DNA methylation of specific loci following developmental exposure to both compounds were observed up to the F2 generation. We identified 2 and 6 out of 10 loci that showed transgenerational effects for MEHP and 5AC, respectively. From the 6 loci that were transgenerationally inherited following 5AC exposures, 5 exhibited the same effect on methylation that was found in sperm samples of F0 fish. Although studies have shown altered methylation patterns in progeny from exposed ancestors, site-specific transgenerational effects that are persistent over generations, as we observe, are rarely reported [47]. For example, in an exposure study of zebrafish adults to either 5AdC, 2,3,7,8-tetrachlorodibenzo-p-dioxin or methylmercury, effects on DNA methylation were observed from F0 livers to F1 zebrafish larvae in methylmercury exposures and no transgenerational effects were observed with any of the exposures [48]. In adult male mice exposed to 5AdC, direct effects on the sperm epigenome were found, but in subsequent generations no effects on DNA methylation were seen [49]. Both studies, however, assessed methylation differences after exposure to adults, which could disrupt methylation in gametes, but misses the sensitive windows of reprogramming events during early development. Furthermore, the mode of action of 5AdC compared to 5AC might differ, since only 10–20% of 5AC will be actively incorporated into the DNA [28]. 5AC shows persistent locus-specific effects spanning generations and suggests that specifically by 5AC, a methylation state can be inherited in zebrafish from one generation to another. Interestingly, the transgenerational effects on methylation of specific loci in F0, F1 and F2 larvae were not consistently reflected in the methylation status of sperm DNA from F0 adults for MEHP. For example, developmental MEHP exposure showed transgenerational hypermethylation of the *cbfa2t2* locus, while F0 sperm showed hypomethylation. This raises the question whether only the paternal methylome acts as a template for DNA methylation, as suggested by others [13, 16]. Alternatively, differential methylation in somatic tissue does not necessarily reflect the methylation status observed in sperm [37]. In contrast to 5AC, we found moderate effects at the 10 analyzed loci following MEHP exposure; however, a few loci exhibited differences in DNA methylation over generations. Early life exposure to DEHP, the parent compound of MEHP, is known to exhibit effects later in life in rodents [19], and transgenerational effects have been found, related to behavior, obesity, early onset of puberty and effects on reproductive organs [24, 50–52]. Furthermore, similar effects on reproductive endpoints have been found in zebrafish after chronic exposure to MEHP, indicating similar modes of action between mammals and zebrafish [53]. Our finding of transgenerational DNA methylation changes in zebrafish exposed to MEHP is also corroborated by other studies in rodents with the DEHP. Transgenerational effects on the sperm methylome have been observed in F3 progeny of F0 pregnant rats exposed to a mixture of plasticizers (DEHP, bisphenol A and dibutylphthalate) [23]. Specifically, over 190 DMRs were found in sperm of F3 progeny; however, no analysis was performed in F2 or F1, which could link these specific DMRs toward transgenerational epigenetically inherited DMRs. A recent study observed moderate intragenerational effects (F0–F1) and no transgenerational effects (F3) on imprinted genes in mice prospermatogonia exposed to 300 mg/kg/day DEHP [25]. However, both studies focused on primordial germ cell reprogramming, since this reprogramming event specifically establishes gender-specific gene imprints in mammals [54]. However, the first wave of reprogramming could be equally essential in epigenetic inheritance as is emphasized by a study in which mice exposed to low doses of DEHP (40 µg/kg/day) directly after fertilization showed effects on DNA methylation at imprinted genes in oocytes up to F2 progeny [26]. In this study, both reprogramming events were included and may explain the strong effects on the imprinted genes up to F2 progeny. These results indicate that not only the reprogramming of primordial germ cells but also the first demethylation wave is of importance in transgenerational epigenetic inheritance.

Many studies have shown the strength of using zebrafish as a vertebrate model to assess DNA methylation [41, 48, 55, 56]. The use of whole zebrafish larvae allows for the assessment of an advanced functional organism for changes in DNA methylation. The drawback of using whole larvae is the complexity of many different cell lineages, each with their own transcriptome and methylome. Small differences in phenotype following exposures may reflect DNA methylation differences due to cell type composition, rather than a compound-specific change in DNA methylation. We cannot completely exclude the possibility that compound exposures may have affected specific cell populations, leading to non-specific effects on DNA methylation. However, to avoid this, we measured DNA methylation in embryos exposed to concentrations that were below the no effect concentration for embryo toxicity. For MEHP, the persistent effects on DNA methylation in subsequent generations were not accompanied by any effects on length. Furthermore, pathways described in previous studies and known to be affected by these compounds were confirmed in our study. Therefore, our results indicate that effects on methylation were directly related to the mode of action of the compound, and not to non-specific effects.

## Conclusions

Developmental exposure of zebrafish embryos to MEHP and 5AC resulted in differential DNA methylation specifically at zfCNEs, suggesting a functional role of DNA methylation on these sequences, conserved throughout evolution. A number of loci that were differentially methylated directly after exposure were also differentially methylated in subsequent generations, indicating transgenerational effects on DNA methylation after developmental exposure to 5AC and MEHP. In the case of 5AC, phenotypic changes were observed in embryo morphology in F1 and F2 progeny. Further research is needed to demonstrate the functional significance of methylation changes in the specific loci studied, as well as genome-wide DMR characterization in F1 and F2 progeny to further map transgenerational effects. Additionally, it is important to link DMRs to histone modifications, and expression of mRNAs and non-coding RNAs to get a view of possible interactions in epigenetic landscapes.

## Methods

### Chemicals

5-Azacytidine (5AC, >98%) was obtained from Sigma (Germany). Mono(2-ethylhexyl) phthalate (MEHP, >99%) was purchased from AccuStandard (USA). Dimethylsulfoxide was purchased from Acros Chemicals (Belgium). The standards 5-methyl-2′-deoxycytidine (mC) and 2′-deoxyguanosine (G) and 5-hydroxymethyl-2′-deoxycytidine (hmC) were purchased at MP biomedicals (the Netherlands), Sigma (Germany) and Carbosynth (UK), respectively. The internal standards for LC/MS analysis (2′-deoxyguanosine-^13^C10,^15^N5 (G-^13^C10^15^N5), 5-methyl-2′-deoxycytidine-d3 (mC-d3) and 5-hydroxymethyl-2′-deoxycytidine-d3 (hmC-d3)) were obtained from Toronto research chemicals (TRC, Canada).

### Zebrafish husbandry

This study was performed in accordance with European Directive 2010/63/EU implemented in the Dutch Act on Animal Experiments. The protocol was approved by The Committee on the Ethics of Animal Experiments of the VU University of Amsterdam under permit number DEC IVM 11-01. All efforts were made to minimize suffering.

Wild-type AB adult zebrafish (*Danio rerio*) were maintained in a recirculation system under a light regimen of 14 h light/10 h dark at a density of 8 fish/L. The parameters of the recirculation system water were: temperature, 26 ± 0.5 °C, pH 7.4 ± 0.2 and conductivity, 525 ± 50 µS. Embryos were obtained by natural mating for an hour of 2 family crosses (6 males:6 females per tank) in 2-L tanks.

### Exposures and transgenerational design of experiment

Fresh stock solutions of 5AC and MEHP were prepared daily in DMSO at concentrations of 10 and 25 mM for 5AC, and 30 mM for MEHP. Embryos (F0) were collected directly after fertilization and immediately transferred to petri dishes containing the chemicals at the final concentration of 0.01% DMSO (1.4 µM), 10 µM 5AC, 25 µM 5AC or 30 µM MEHP in medium (294 mg/L CaCl_2_, 123 mg/L MgSO_4_, 64.7 mg/L NaHCO_3_, 5.7 mg/L KCl), allowing exposures directly after fertilization. Fertilized and good quality eggs were selected under a stereomicroscope (M7.5 Leica, Eindhoven, the Netherlands), and randomly transferred to a 24-well suspension culture plate in pools of 12 embryos/well containing 2 mL of exposure medium, and maintained in an incubator on a 14-h light/10-h dark cycle at 26 °C. Each day, embryos (from 1 to 6 dpf) were checked for developmental malformations and 90% exposure medium was refreshed with freshly made exposure medium. After 6 days, the exposure was terminated, and pools of zebrafish larvae were collected for RNA and DNA analysis (Table 3) and a total of 120 larvae were equally divided over 4 tanks containing 300 mL medium per treatment. Feeding of 2.3 mg powdered baby food (Sera micron) and 2 mL of *Tetrahymena* suspension twice a day was started at this time point. At 15 dpf, one tank of fish was used to for DNA and RNA analysis (Table 3). The fish from the other three tanks were transferred to a 2-L tank and maintained in the recirculation system. Fish were fed twice with 6 mg powdered feed (Tetraminbaby/Tetrapro) and 5 mL of *Tetrahymena* suspension and once with 5 droplets of Artemia suspension a day per tank. From 20 dpf on, fish were fed with 6 mg powdered feed and 5 mL Artemia suspension. After 23 weeks, fish were able to produce enough eggs for subsequent analyses and the establishment of F1 generation. From each tank, 8 males and 12 females were crossed in 2 separate groups, and eggs were collected, and after 24 h pooled similarly as described above. From each tank, replicate pools of larvae were taken for RNA and DNA analysis at 6 dpf (Table 3) and from each tank 80 larvae were equally divided over 2 × 300 mL tanks. At 15 dpf one tank was used for DNA analysis (Table 3) and the fish from the other tanks were transferred to 2-L tanks. Similar as above, F1 was raised and mated at 23 weeks of age to generate F2. F2 was raised until 6 dpf, when larvae were collected for further analysis. See Fig. 1 for details.

**Table 3:** Number of biological replicates analyzed per exposure

Samples per exposure group	Gen	Length	DNA^a^	RNA^a^	Global methylation^b^	RRBS^b^	BisPCR2^b^
3 dpf Larvae	F0	68–76					
6 dpf Larvae	F0	50–60	6	5	6	5	6
15 dpf Larvae	F0	10	5		5		5
Brain per gender	F0		15		15		
Liver per gender	F0		15		15		
Sperm	F0		15		15		6
6 dpf Larvae	F1	30	9		9		5
15 dpf Larvae	F1	30	9		9		
6 dpf Larvae	F2	30	9		9		5

^a^Pool of 10 or 2 larvae for 6 or 15 dpf, respectively

^b^Number of biological replicates used in specific methylation analysis

### Length analysis

Images from hatched zebrafish (3, 6 and 15 dpf) were analyzed by ImageJ. From each time point at each generation, the difference in length was calculated, and a two-tailed *t* test was performed with a Bonferroni correction for multiple comparisons per exposure, comparing the different generations.

### Gene expression analysis

RNA was purified using the Nucleospin total RNAII extraction kit (Macherey–Nagel, Germany). RNA was extracted from pools of 10 or 2 larvae (6 or 15 dpf, respectively) (Table 3). Larvae were collected in tubes with ceramic beads and snap-frozen in liquid nitrogen. RA1 lysismix (Macherey–Nagel, Germany) was added to the tubes and samples were homogenized using Precellys homogenization (Precellys, USA), followed by RNA extraction according the manufacturers’ protocol. Equal amounts of RNA were reverse transcribed with the high-capacity cDNA RT kit (Applied Biosystems, Grand Island, NY), followed by a 10 times dilution of the cDNA reaction with Milli-Q water. QPCRs on the diluted cDNA were performed in 10 µL, containing 5 µL Lightcycler 480 SYBR Green I Master mix (Roche, Norway), 250 nM of forward and reverse primers, 2 µL diluted cDNA and Milli-Q water, in technical duplicates. PCR was performed on a Roche Lightcycler 96 (Roche, Norway), with 5-min denaturation at 95 °C, followed by 40 cycles of 15 s at 95 °C and 45 s at 60 °C. After the run a melting curve was generated from 60 to 90 °C. Primers for reference genes were developed using the Primer-BLAST software from NCBI (http://www.ncbi.nlm.nih.gov/) (Additional file 1: Table 3). *Dnmt* primer sequences were obtained from a recently published study [57] (Additional file 1: Table 3). All primers were validated for specificity by melting curve analysis and gel electrophoresis. Efficiency of primers was determined against a dilution curve of pooled zebrafish cDNA. Cq values were determined using linreg [58]. Five reference genes were measured (*hprt1*, *ef1a*, *beta*-*actin*, *hmbs* and *rps18*), of which *ef1a* and *hprt1* were most stable and were used for relative gene expression calculations, using the geometric average of two reference genes as described earlier [59]. Statistical analysis was performed in GraphPad 5.04 (GraphPad Software Inc., La Jolla, CA). For statistics, relative gene expression was log2-normalized and ANOVA was performed per gene, with Dunnets’ post hoc tests for multiple comparisons.

### DNA purification

Precipitation and purification of genomic DNA was performed with the Gentra puregene tissue DNA extraction kit (Qiagen, Germany). Pools of zebrafish (Table 3) were snap-frozen in liquid nitrogen. Prior to DNA extraction the pools of zebrafish larvae were disrupted in lysis buffer with a 20G needle. DNA was extracted as described earlier [29]. Quality of DNA was assessed by gel electrophoreses for fragmentation and RNA contamination, and quantity and purity by NanoDrop (ND-1000, Thermo Scientific, Germany).

### Global methylation analysis

Analysis of mC and hmC relative against G was analyzed with liquid chromatography–tandem mass spectrometry (LC/MS, Agilent 6490) as described previously [29], with modifications. To account for fluctuations between LC runs, we adapted the protocol with the inclusion of labeled internal standards for each of the analytes. Genomic DNA (200 ng in 10 µL E buffer) was digested by adding 10 µL of a mixture of benzonase, phosphodiesterase and alkaline phosphatase (50 U/mL, 60 mU/mL and 40 U/mL, respectively (Sigma, Germany) in buffer (20 mM TRIS, 100 mM NaCl and 20 mM MgCl_2_, pH 7.9). A standard curve was included with percentages that were expected in zebrafish (0–12% mC and 0–1% hmC), based on molarity of mC and hmC relative to G (345 nM in 200 µL). Following incubation of 6 h at 37 °C, a mix of internal standards was added to the samples and standards, with final concentrations of 345, 20 and 0.69 nM for G-^13^C10^15^N5, mC-d3 and hmC-d3, respectively, in a final volume of 200 µL. For LC/MS analysis, the same mass transitions were used as described earlier [29], but with extra mass transitions of 283.3/167.2 for ^13^C10^15^N5-G, 261.1/145.1 for hmC-d3 and 245.1/129.1 for mC-d3. Ionization-specific parameters were similar for all compounds; dwell time 50 ms, collision energy 2 V, fragmentor 380 V, cell accelerator voltage 4. QQQ-specific parameters were a gas temperature of 200 °C, gas flow of 14 L/min, nebulizer gas at 45 psi, sheath gas temperature of 350 °C, sheath gas flow of 7 L/min, capillary voltage of 3000 V (positive mode), nozzle voltage of 500 an iFunnel parameters of high-pressure RF of 150 psi and low-pressure RF of 60 psi. For all samples and calibration curves, we first calculated the ratio in peak areas of mC/mC-d3 (ratio mC), hmC/hmC-d3 (ratio hmC) and G/G-^13^C10^15^N5 (ratio G), followed by (ratio mC/ratio G) and (ratio hmC/ratio G). For the standard curve, these ratios were plotted against the percentage (hydroxy)methylation (0–12% for mC and 0–1% for hmC). We added a quality control (QC) sample to every series to calculate the deviation between experiments. This QC sample consisted of a pool of 48 hpf zebrafish DNA. All samples were interpolated in the calibration curves. Statistical significance was performed with ANOVA, using Dunnets’ multiple comparison post hoc tests within GraphPad software 5.04 (Inc., La Jolla, USA).

### Reduced representation bisulfite sequencing (RRBS)

Genomic DNA was measured with the Quant-it Picogreen dsDNA assay (Thermo Fischer) and 1 µg total genomic DNA was digested overnight with MspI at 37 °C (NEB, USA). Digestions were terminated by adding 0.5 M EDTA, and digested DNA was purified on a GeneJET PCR purification column (Thermo Fischer). A library was made using the NEBNext Ultra DNA library preparation kit for Illumina, including methylated index adapters. Adapter ligated fragments were bisulfite converted using the Zymo EZ DNA Methylation Gold kit (Zymo Research, USA), followed by 14 cycles of PCR. PCR products were purified using AMPure XP beads and quality was assessed on a Bioanalyzer (Agilent, Belgium), using a high-sensitivity DNA chip (Agilent, Belgium). Concentration of the library was measured by QPCR. Samples were pooled in equal concentrations and sequencing was performed on an Illumina HiSeq 2500 in paired end (2 × 100 bp), according to the manufacturers’ recommendations for RRBS sequencing. Data analysis was performed using a bisulfite analysis pipeline, developed by Babraham institute (UK). Detailed procedures are provided in supplementary materials and methods (Additional file 2). In short, after sequencing, Fasta files were first adapter and quality trimmed with Trim_galore (version 0.4.0, Babraham bioinformatics), followed by Bismark alignment (version 0.14.5, Babraham bioinformatics, [30]) to the recently released zebrafish genome assembly GRCz10. Downstream analysis was performed with the methylKit package in R (version 3.2.2), using logistic regression analysis with a sliding linear model to correct for multiple comparisons, using Benjamini–Hochberg false discovery rate (FDR) correction (Q-value) [60]. Within methylKit, our approach was to detect differentially methylated regions (DMRs) by dividing the genome in 300-bp tiles containing at least 4 mutually covered Cs in CpG context, with at least 5 reads per C, resulting in ≥20 observations in each replicate, while controlling the FDR at 0.01 and using a methylation difference cutoff of 10%. The use of 5 pooled replica’s per treatment, a sufficient read depth over multiple CpG sites, and the use of logistic regression, combined with an FDR approach should account for sampling bias. A second approach was to identify specific differentially methylated CpG sites (DMCs) for downstream locus-specific analysis. We used logistic regression analysis on Cs in CpG context with at least 10 reads, with FDR controlled at 5% and a difference cutoff of 20%.

We used the DMRs in the Seqmonk genome browser (version 0.32) to investigate enrichment on different features, gene promoters, gene bodies, CpG islands and shores. Gene promoters were defined as 2000-bp upstream of a transcriptional start site (TSS). CpG islands were calculated according to the Takai and Jones algorithm [61]. We adjusted the parameters because of the different GC content and observed/expected ratio of CpG sites in zebrafish compared to mammals (CG > 0.45, o/e > 0.65), and defined shores as 2000-bp regions surrounding the CpG islands. Furthermore, we used a list of zebrafish conserved non-genic elements (zfCNEs) [35] and regions with developmental enhancer marks [12] to calculate enrichment on these specific elements.

### Pathway analysis

Associated genes from DMRs were predicted using Genomic Regions Enrichment of Annotations Tool (GREAT) [62]. This tool predicts gene functions of cis-regulatory elements in a genome. We took the standard parameters used in GREAT to predict functional genes (5000-bp upstream and 1000-bp downstream of a TSS, with an extension of max 1 Mb to the next regulatory domain of the nearest gene). The resulting gene list was used in Ingenuity Pathway Analysis (IPA, QIAGEN Redwood City, www.qiagen.com/ingenuity). IPA uses canonical pathways based on human, mice and rat data, but is able to import and analyze zebrafish gene homologs, based on mammalian knowledge bases. Since there is no apparent relationship between genes expressed and the methylation state of a regulatory region, we did not provide the methylation difference as an extra parameter in IPA. We used IPA to predict upstream regulatory features and to search for enrichments in toxicology and disease gene lists. IPA analysis was performed using Fischer’s exact test and *P* values <0.05 were considered significant.

### BisPCR2

For validation of RRBS data and analysis of DMCs in F0 15 dpf, sperm and subsequent 6 dpf generations (F1 and F2), we used a recently developed method by Bernstein et al. [32]. Detailed procedures are presented in supplementary materials and methods (Additional file 2). BisPCR2 uses two rounds of PCR on bisulfite converted DNA. One round is used for amplifying bisulfite converted DNA using specific primers with Illumina adapter overhangs. After PCR#1, all amplicons of one sample are pooled in equal amounts and subjected to a second PCR using the standard Illumina library and index primers as reported by Bernstein et al. [32]. From the single CpG analysis from the RRBS results, we initially selected 10 Cs in loci which were differentially methylated in either 5AC or MEHP exposures (Additional file 1: Table 4). We validated this method using a calibration curve of control DNA from 0 to 100% methylated DNA for efficiency assessment of the PCRs and technical variation using two samples that were assessed in duplicate. Sequencing was performed on an Illumina MiSeq sequencer (Illumina Inc., USA) as described by Bernstein et al. Mapping of sequences and statistics was performed similarly as with RRBS analysis.

## Additional files

**Additional file 1.** A Microsoft Word document that contains the additional Tables and Figures as cited in the main text.

**Additional file 2.** A Microsoft Word document that contains detailed descriptions of used bioinformatics and the BisPCR2 method.

**Additional file 3.** A Microsoft Excel spreadsheet that contains all the DMRs and IPA output for MEHP.

**Additional file 4.** A Microsoft Excel spreadsheet that contains all the DMRs and IPA output for 5AC.

## Abbreviations

MEHP: mono(2-ethylhexyl) phthalate
5AC: 5-azacytidine
dpf: days post-fertilization
hpf: hours post-fertilization
mC: methyl cytosine
DNMT: DNA methyltransferase
PGC: primordial germ cell
DEHP: di-2-(ethylhexyl) phthalate
DMR: differentially methylated region
hmC: hydroxymethyl cytosine
LC/MS: liquid chromatography–tandem mass spectrometry
BisPCR2: multiplex loci-specific bisulfite sequencing
DMC: differentially methylated CpG
TSS: transcriptional start site
CGi: CpG island
zfCNE: zebrafish conserved non-genic elements
GREAT: Genomic Regions Enrichment of Annotations Tool
IPA: ingenuity pathway analysis
TGF: transforming growth factor
SSH: slingshot protein phosphatase
SOX: SRY (sex determining region Y)-box
POU: POU domain
PPAR: peroxisome proliferator-activated receptor
RXR: retinoic X receptor
ASCL: achaete-scute family bHLH transcription factor
PAX: paired box
GLI: GLI family zinc finger
KLF: kruppel-like factor
HNF: hepatocyte nuclear factor 4
RRBS: reduced representation bisulfite sequencing
myc: v-myc avian myelocytomatosis viral oncogene homolog
nrp: neuropilin
cbfa: core-binding factor, runt domain
cps: carbamoyl-phosphate synthase
gabr: gamma-aminobutyric acid (GABA) A receptor
