# Supplementary material for: Differential DNA methylation at conserved non-genic elements and evidence for transgenerational inheritance following developmental exposure to mono(2-ethylhexyl) phthalate and 5-azacytidine in zebrafish
Source: Epigenetics Chromatin. 2017 Apr 12;10:20. doi: 10.1186/s13072-017-0126-4 (PMC5389146; doi:10.1186/s13072-017-0126-4)
Supplement: Supplementary file 1 — Additional file 1. A Microsoft Word document that contains the additional Tables and Figures as cited in the main text. [file 13072_2017_126_MOESM1_ESM.docx]

Additional file 1:

Table 1: Mapping results RRBS following Bismark alignments and MethylKit analysis

|  |  | Mapping Efficiency | %C in CpG | %C in CHG | %C in CHH | bisulfite conversion efficiency* | Mapped regions | Mapped CpG 10r |
| --- | --- | --- | --- | --- | --- | --- | --- | --- |
| Sample_844 | Control1 | 60.2 | 77.9 | 1.0 | 0.7 | 99.26 | 120145 | 542585 |
| Sample_845 | Control2 | 59.3 | 77.9 | 1.0 | 0.7 | 99.26 | 150754 | 482559 |
| Sample_846 | Control3 | 59.8 | 77.3 | 1.0 | 0.7 | 99.26 | 152279 | 486403 |
| Sample_847 | Control4 | 58.3 | 76.7 | 1.0 | 0.7 | 99.20 | 157704 | 495952 |
| Sample_848 | Control5 | 59.5 | 77.7 | 1.0 | 0.7 | 99.27 | 143643 | 467583 |
| Sample_849 | MEHP1 | 59.2 | 77.3 | 1.0 | 0.7 | 99.27 | 113306 | 368262 |
| Sample_850 | MEHP2 | 59.5 | 77.9 | 1.0 | 0.7 | 99.27 | 126368 | 417905 |
| Sample_851 | MEHP3 | 59.9 | 77.9 | 1.0 | 0.7 | 99.28 | 139910 | 454898 |
| Sample_852 | MEHP4 | 59.0 | 77.0 | 1.1 | 0.7 | 99.20 | 139808 | 453697 |
| Sample_853 | MEHP5 | 59.3 | 77.3 | 1.0 | 0.7 | 99.19 | 144503 | 466496 |
| Sample_854 | 5AC1 | 59.6 | 77.7 | 1.0 | 0.7 | 99.27 | 143028 | 466040 |
| Sample_855 | 5AC2 | 59.2 | 77.0 | 1.0 | 0.7 | 99.28 | 101961 | 312047 |
| Sample_856 | 5AC3 | 58.4 | 76.7 | 1.0 | 0.7 | 99.21 | 121503 | 403161 |
| Sample_857 | 5AC4 | 58.8 | 76.9 | 1.0 | 0.7 | 99.20 | 133817 | 445084 |
| Sample_858 | 5AC5 | 59.8 | 77.8 | 1.0 | 0.7 | 99.28 | 126292 | 410153 |
| Average |  | 59.3 | 77.4 | 1.0 | 0.7 | 99.25 | 134335 | 444855 |

*Assessed by MethylKit

Table 2: Overview of the total amount of tiles at different features versus the amount of differentially methylated tiles (DMRs). (A) MEHP specific tiles and DMRs. (B) 5AC specific tiles and DMRs. * indicates enrichment outside the analyzed feature

| 1. MEHP | Total tiles | DMRs | p hyper |
| --- | --- | --- | --- |
|  | 66896 | 410 |  |
| Promoters | 2547 | 12 | 8.22E-01 |
| Gene bodies | 38906 | 290 | 7.54E-04* |
| CGis | 42147 | 185 | 7.58E-01 |
| CNEs | 2514 | 80 | 1.25E-31 |

| 1. 5AC | Total tiles | DMRs | p hyper |
| --- | --- | --- | --- |
|  | 61455 | 580 |  |
| Promoters | 2357 | 19 | 2.12E-01 |
| Gene bodies | 35624 | 290 | 5.06E-05* |
| CGis | 39288 | 185 | 3.20E-54* |
| CNEs | 2296 | 80 | 5.20E-23 |

Table 3: Primer sequences used for RT QPCR

| **DNMTs** | **Forward** | **Reverse** | **Accession number** |
| --- | --- | --- | --- |
| ef1a | TTGAGAAGAAAATCGGTGGTGCTG | GGAACGGTGTGATTGAGGGAAATTC | NM_131263.1 |
| bactin | CGAGCAGGAGATGGGAAC | CAACGGAAACGCTCATTGC | NM_131031.1 |
| rps18 | CATCCCAGAGAAGTTTCAGCACATC | CGCCTTCCAACACCCTTAATAGC | NM_173234.1 |
| hmbs | GTGTGTGGAATTGGACAACAAAGTG | CGAGGGCTGATGATGAGATATTGC | NM_201154.1 |
| hprt1 | CAGCGATGAGGAGCAAGGTTATG | GTCCATGATGAGCCCGTGAGG | NM_212986.1 |
| dnmt1* | GAGCCTGTGAAGCAGGAGAA | CATGAATGGCACTGCACAGA | NM_131189.2 |
| dnmt3bb.2* | AAACAACGCGCTTCCACG | TTCCATAACCACCACCGTCC | AF135438.1 |
| dnmt3bb.1* | GCGTCAGAAGTATGCGAGGA | GACCTTTCCTAGCAGGGTTGA | AB196915.1 |
| dnmt3bb.3* | GCTCCATCACATCTCAGCCC | CAAATCCGACACCGGCAAAG | XM_009296722.1 |
| dnmt3ab* | AGAAAACCCATTCGCGTCCT | GTGCCCTCGTAGAGACCTTTT | AB196917.1 |
| dnmt3ba* | ATCCGACATCTCTTTGCACC | GTGAAGTGAATTTGCAGAAAGC | AB196918.1 |
| dnmt3aa* | GGACGTATTGTGTCCTGCT | ATCACCAAACCACATGACCC | AB196919.1 |

*sequences taken from Santangeli et al. 2016

Table 4: Primer sequences used for BisPCR2 analysis

|  | | associated gene | Location from TSS | Meth diff (%) | Region for validation | no. CpG | Strand | Ta | Ratio  PCR#2 |
| --- | --- | --- | --- | --- | --- | --- | --- | --- | --- |
| CvsMEHP DMC | |  |  |  |  |  |  |  |  |
| Chr2:43611806-43611806 | | nrp1b | 8023 upstream | 25 | Chr2:43611512-43611846 | 6 | -1 | 52 | 2 |
| Primer F | nrp1b_F | ACACTCTTTCCCTACACGACGCTCTTCCGATCTGAGAGTTAGTTTGTTTAGATATT | | | | | | |  |
| Primer R | nrp1b_R | GTGACTGGAGTTCAGACGTGTGCTCTTCCGATCTCTTCCATAACCCTTTATATT | | | | | | |  |
| Chr4:53831128-53831128 | | CT583728.4 | 399 upstream | 24 | Chr4:53831094-53831415 | 16 | 1 | 54 | 2 |
| Primer F | CT583728.4_F | ACACTCTTTCCCTACACGACGCTCTTCCGATCTTTAAAAAGTATGGAAGGTAAGTG | | | | | | |  |
| Primer R | CT583728.4_R | GTGACTGGAGTTCAGACGTGTGCTCTTCCGATCTCCCATTCTACTTCAACATAATA | | | | | | |  |
| Chr9:39356710-39356710 | | cps1 | 399 upstream | -24 | Chr9:39356640-39356899 | 11 | 1 | 52 | 2 |
| Primer F | cps1_F | ACACTCTTTCCCTACACGACGCTCTTCCGATCTTTGTTAAGTGTTTGTGATTGT | | | | | | |  |
| Primer R | cps1_R | GTGACTGGAGTTCAGACGTGTGCTCTTCCGATCTAACTAACCTATCTAATTATTCCC | | | | | | |  |
| Chr12:29105853-29105853 | | Gabrz | 165848 downstream | 25 | Chr12:29105587-29105921 | 13 | -1 | 50 | 2 |
| Primer F | gabrz_F | ACACTCTTTCCCTACACGACGCTCTTCCGATCTTTTTTATTTTTATGAAGGTTTTG | | | | | | |  |
| Primer R | gabrz_R | GTGACTGGAGTTCAGACGTGTGCTCTTCCGATCTAAACCTAACCTATTTAAATC | | | | | | |  |
| Chr20:43514811-43514811 | | si:dkey-14a7.6 | 1531 upstream | -26 | Chr20:43514527-43514862 | 13 | -1 | 50 | 2 |
| Primer F | si:dkey-14a7.6_F | ACACTCTTTCCCTACACGACGCTCTTCCGATCTATTAAGTTTAGAGTGTGGAA | | | | | | |  |
| Primer R | si:dkey-14a7.6_R | GTGACTGGAGTTCAGACGTGTGCTCTTCCGATCTAACCCTACTAAAATAAATTATAT | | | | | | |  |
| Chr21:20158965-20158965 | | si:dkey-247m21.3 | 34401 upstream | 30 | Chr21:20158921-20159221 | 7 | 1 | 52 | 2 |
| Primer F | si:dkey-247m21.3_F | ACACTCTTTCCCTACACGACGCTCTTCCGATCTTGATGTGAGATTGGTTATTT  GTGACTGGAGTTCAGACGTGTGCTCTTCCGATCTCAAACATAACTTCAATACTC | | | | | | |  |
| Primer R | si:dkey-247m21.3_R |  |  |  |  |  |  |  |  |
| Cvs5AC DMC | |  |  |  |  |  |  |  |  |
| Chr2:32025720-32025720 | | mycb | 7318 upstream | 34 | Chr2:32025720-32025720 | 7 | 1 | 54 | 7 |
| Primer F | mycb_F | ACACTCTTTCCCTACACGACGCTCTTCCGATCTGAAATAAGATATTTTAAAGTGTT | | | | | | |  |
| Primer R | mycb_R | GTGACTGGAGTTCAGACGTGTGCTCTTCCGATCTACAATAACACTAACACCAAA | | | | | | |  |
| Chr3:48276633-48276633 | | si:ch211-245b21.1 | 1957 upstream | -30 | Chr3:48276549-48276951 | 12 | -1 | 56 | 2 |
| Primer F | si:ch211-245b21.1_F | ACACTCTTTCCCTACACGACGCTCTTCCGATCTTAGGATAGAAGATAGTTAGAAGT | | | | | | |  |
| Primer R | si:ch211-245b21.1_R | GTGACTGGAGTTCAGACGTGTGCTCTTCCGATCTTTTTATCCAAAAATACCAATACT | | | | | | |  |
| Chr6:57641668-57641668 | | cbfa2t2 | 84319 gene body | 32 | Chr6:57641533-57641785 | 14 | 1 | 56 | 7 |
| Primer F | cbfa2t2_F | ACACTCTTTCCCTACACGACGCTCTTCCGATCTTAGGTTTTTTAGGGTGTATT | | | | | | |  |
| Primer R | cbfa2t2_R | GTGACTGGAGTTCAGACGTGTGCTCTTCCGATCTTATATTTATATTCATCTTCAATC | | | | | | |  |
| Chr25:36706591-36706591 | | si:dkey-234i14.6 | 8250 gene body | -36 | Chr25:36706556-36706894 | 15 | -1 | 54 | 7 |
| Primer F | si:dkey-234i14.6_F | ACACTCTTTCCCTACACGACGCTCTTCCGATCTTAATTAAAAATTGGATGTTATT | | | | | | |  |
| Primer R | si:dkey-234i14.6_R | GTGACTGGAGTTCAGACGTGTGCTCTTCCGATCTAAATCTCTAAAAATATTCATCT | | | | | | |  |

Figure 1: Average standard deviation of all CpGs analyzed with BisPCR2 within technical replicates. 5AC5 and 5AC6 represent the two different samples that were analyzed for technical variation. Error bars represent SEM.

Figure 2: Calibration curves from BisPCR2 analysis. Calibration curves represent average methylation off all CpG sites on a specific locus.

Figure 3: Correlation plot of mutual analyzed CpG sites between RRBS and BisPCR2 analysis. (Spearman correlation = 0.8885, P <0.0001)

Figure 4: Phenotypic effects on swim bladder and gastrointestinal tract in 5-azacytidine (5AC) exposed larvae. Pictures represent individual larvae at 15 dpf.


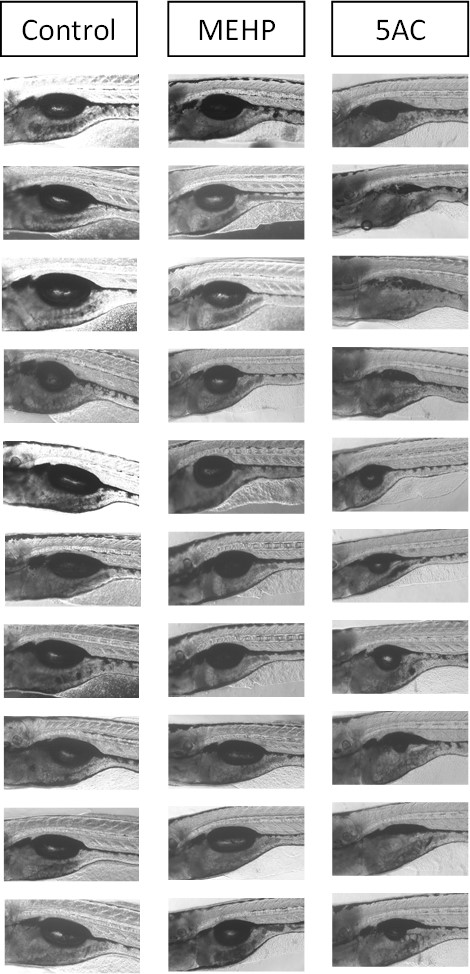


Figure 5: Global methylation analysis of zebrafish larvae exposed to 5-azacytidine (5AC) from 0 to 6 dpf (n=6, Error bars represent 95% CI, ANOVA with Dunnets multiple comparisons P < 0.05).

Figure 6: Cluster analysis (Ward) of DNA methylation after exposure of zebrafish larvae exposed to 10 and 25 µM 5-azacytidine (5AC) from 0 to 6 dpf. Highlighted are clusters that represent dose response relations.

Figure 7: Hierarchical clustering of methylation measured in 10 specific loci in F0 larvae 6 dpf and 15 dpf, and adult (sperm) samples, as well as F1 and F2 larvae (6dpf) (Ward clustering).
