# Supplementary material for: Differential DNA methylation at conserved non-genic elements and evidence for transgenerational inheritance following developmental exposure to mono(2-ethylhexyl) phthalate and 5-azacytidine in zebrafish
Source: Epigenetics Chromatin. 2017 Apr 12;10:20. doi: 10.1186/s13072-017-0126-4 (PMC5389146; doi:10.1186/s13072-017-0126-4)
Supplement: Supplementary file 2 — Additional file 2. A Microsoft Word document that contains detailed descriptions of used bioinformatics and the BisPCR2 method. [file 13072_2017_126_MOESM2_ESM.docx]

Differential DNA methylation at conserved non-genic elements and evidence for transgenerational inheritance following developmental exposure to mono(2-ethylhexyl) phthalate and 5-azacytidine in zebrafish

Jorke H Kamstra^1*^, Liana Bastos Sales^2^, Peter Aleström^1^ and Juliette Legler^2 3^

Affiliations

^1^Faculty of Veterinary Medicine and Biosciences, Dept. of Basic Science and Aquatic Medicine, CoE CERAD, Norwegian University of Life Sciences, P.O. Box 8146 Dep., 0033 Oslo, Norway, [jorke.kamstra@nmbu.no](mailto:jorke.kamstra@nmbu.no), [peter.alestrom@nmbu.no](mailto:peter.alestrom@nmbu.no)

^2^Institute for Environmental Studies, VU University Amsterdam, Amsterdam, The Netherlands, [liana.bastossales@vu.nl](mailto:liana.bastossales@vu.nl)

^3^Institute for Environment, Health and Societies, College of Health and Life Sciences, Brunel University London, Uxbridge, United Kingdom, [juliette.legler@brunel.ac.uk](mailto:juliette.legler@brunel.ac.uk)

Supplemental methods

Mapping

Sequences were mapped using the genome assembly GRCz10. Trimming was performed with trim_galore (v0.4.0) with the following parameters:

trim_galore --quality 20 --phred64 --paired --three_prime_clip_R1 10 --clip_R2 5 --three_prime_clip_R2 10 --fastqc --rrbs

Mapping of bisulfite converted DNA was performed with Bismark (v0.14.5 [1]), with bowtie2 (v2.2.6). Since standard mapping parameters generated low mapping efficiency (~30%), likely to the difference in strains between the AB strain we used and the assembled tuebingen line from GRCz10, we used slightly relaxed parameters for mapping. This resulted in efficiencies of around 59 % (Additional file 1, table 3). Bismark mapping was performed with the following parameters:

bismark --bowtie2 -N 0 -L 20 --score_min L,0,-0,6

In the final bismark_methylation_extractor C in CpG, CHH and CHG were extracted with the parameter – no_overlap, to ensure that overlapping reads from read 2 were not measured twice in the final analysis.

Analysis with MethylKit [2].

Sorted bismark.sam files were imported in MethylKit with the read.bismark function, only allowing sequence reads with a phred score higher than 20 and minimum 5 reads per CpG. Next, samples were filtered by excluding reads above the 99.9 percentile, since these reads are either mapped against repeat elements or have very high PCR bias.

Two strategies were taken to assess differential methylation. First using MethylKit, differentially methylated regions (DMRs) were found by dividing the genome in regions of 300 bp, with at least 4 Cs analyzed with at least 5 reads, following logistic regression analysis with a sliding linear model for false discovery rate correction (FDR). With this analysis, DMRs were detected using a FDR cut off of 1 % and a methylation difference of at least 10 %. These probes were used for downstream pathway analysis (see additional files 3 and 4). In subsequent analyses the DMR table was imported in the Seqmonk (v0.32, Babraham bioinformatics) genome viewer. The DMRs were mapped against different features (e.g. promoters, gene bodies, CNEs).

To validate RRBS data and search for transgenerational effects with locus specific analysis, a second analysis was performed in Seqmonk (v0.32, Babraham bioinformatics) with the logistic regression filter, by analyzing single CpGs (DMCs) with at least 10 reads, using a FDR cut off of 5 % and a methylation difference of 20 %. These probes were used for validating RRBS data and search for transgenerational effects with loci specific analysis.

Validation set and transgenerational assessment of loci with BisPCR2.

We developed primers for a subset of DMCs. We used the recently developed BisPCR2 method which uses two rounds of PCR [3]. One round is used for amplifying bisulfite converted DNA using primers with Illumina adapter overhangs (PCR#1). In a second PCR all amplicons of one sample are pooled in equal amounts (gel electrophoresis intensity bands) and are subjected to PCR with specific indices and standard Illumina library primers (PCR#2). Bernstein et al reported 48 use of primer indices in their method. We followed the method with slight deviations [3]. First we developed primers using Bisearch (<http://bisearch.enzim.hu/>), added the overhangs and optimized and checked the products with the type-it HRM kit (Qiagen, Germany). We checked the right product size, the Cq value of the product and the efficiency of the PCR with unmethylated to methylated control material. To produce unmethylated DNA, we subjected 50 ng of 6 dpf zebrafish genomic DNA to a whole genome amplification reaction with the repli-G kit (Qiagen, Germany) according to the manufacturer’s recommendations. Furthermore we subjected 1 µg of the same DNA sample to an M.SssI CpG methylase reaction (New England Biolabs, USA) to produce fully methylated DNA using 2 µL of NEB2 buffer, 0.5 µL of 32 mM SAM, 4 U of M.SssI in a 20 µL reaction. After 2 hrs incubation at 37 ºC another 0.5 µL of SAM and 4 U of M.SssI was added to the sample and the mixture was incubated for another 4 hrs. All DNA from controls and experiments were bisulfite converted with the Epitect bisulfite kit according to the manufacturer (Qiagen, Germany) and measured on Nanodrop (ND-1000, Thermo Scientific) in ssDNA mode for an estimation of the concentration. All samples were diluted to 5 ng/µL and control bisulfite converted DNA was mixed in different ratios (0, 25, 50, 75 and 100 %). Type-IT HRM reactions, which is the same family of TAQ as the Pyromark kit (used in the BisPCR2 method), were performed in 10 µL buffer, 700 nM primer mix and 10 ng template in 20 µL reactions. All PCR reactions were performed with a 5 min hot start and cycling condition of 95 ºC for 30 sec, optimized Ta for 30 sec, and extension at 63 ºC for 1 min (45 cycles). The optimized Ta is shown in additional file 1 table 3. Following optimization, the protocol of Bernstein et al was followed using the Pyromark PCR kit (Qiagen, Germany), using optimized annealing temperatures and an extension temperature of 63 ºC, which enhanced Cq values and unbiased the PCR reactions with regard to methylated or unmethylated DNA. PCR#1 reactions were performed with the Pyromark PCR kit (Qiagen, Germany) in 20 µL reactions (10 µL buffer, 700 nM primer mix, 20 ng DNA template) with a 15 min hot start, followed by 45 cycles of 95 ºC 30s, Ta ºC 30s and 63ºC for 1 min and a final extension of 10 min. After all PCR reactions were performed, amplicons were pooled by sample. PCR#1 pools were purified using the Qiagen PCR purification kit (Qiagen, Germany) and concentrations were measured on Nanodrop (ND-1000, Thermo Scientific). PCR#2 reactions were performed with index specific primers in 20 µL reactions consisting of 10 µL pyromark buffer, 700 nM index primer, 700 nM library primer and 1 ng of pooled PCR#1 product, with 15 min hot start, followed by 10 cycles of 95 ºC 30 s, 56 ºC 30 s and 63 ºC for 2 min followed by a final extension of 10 min. PCR#2 samples were purified with AMPure XP beads (Beckman Coulter, USA) according to manufacturer. Concentrations were measured using the Qubit fluorimeter (Life technologies, Norway) and calculated to nM using the formula:

c (nM) =(Qubit conc (ng/µL)*1000000)/(660*average length)

A subset of PCR#2 samples was analyzed on Agilent 2100 bioanalyzer and highly comparable profiles were observed between analyzed samples (data not shown). PCR#2 samples were diluted in equimolar portions to 2 nM and next generation sequencing was performed as described earlier by Bernstein et al [3]. After sequencing the samples were analyzed as described above for RRBS. Differential methylation was calculated using logistic regression with BH FDR multiple comparisons and sites with a difference of more than 10% methylation were considered biologically relevant differences.

Literature

1. Krueger F, Andrews SR. Bismark: A flexible aligner and methylation caller for Bisulfite-Seq applications. Bioinformatics. 2011;27:1571–2.

2. Akalin A, Kormaksson M, Li S, Garrett-Bakelman FE, Figueroa ME, Melnick A, et al. methylKit: a comprehensive R package for the analysis of genome-wide DNA methylation profiles. Genome Biol. 2012;13:R87.

3. Bernstein DL, Kameswaran V, Le Lay JE, Sheaffer KL, Kaestner KH. The BisPCR2 method for targeted bisulfite sequencing. Epigenetics Chromatin. 2015;8:27.
